# Solving the Enigma of the Identity of *Laccaria laccata*

**DOI:** 10.3390/jof11080575

**Published:** 2025-08-01

**Authors:** Francesco Dovana, Edoardo Scali, Clarissa Lopez Del Visco, Gabriel Moreno, Roberto Para, Bernardo Ernesto Lechner, Matteo Garbelotto, Tom W. May

**Affiliations:** 1 Dipartimento di Bioscienze, Biotecnologie e Ambiente (DBBA), Università degli Studi di Bari “Aldo Moro”, Via Orabona 4, 70125 Bari, Italy; 2 Department of Environmental Science, Policy and Management, University of California, 54 Mulford Hall, Berkeley, CA 94720, USA; edoardo_scali@berkeley.edu (E.S.); c-lopez-delvisco@berkeley.edu (C.L.D.V.); matteog@berkeley.edu (M.G.); 3 Department of Life Sciences (Botany), Biology Building, University of Alcalá, 28805 Alcalá de Henares, Spain; gabriel.moreno@uah.es; 4 Via Martiri di via Fani 22, 61024 Mombaroccio, Italy; r.para@alice.it; 5 Laboratorio de Hongos Agaricales, Departamento de Biodiversidad y Biología Experimental, Facultad de Ciencias Exactas y Naturales, Universidad de Buenos Aires, Buenos Aires 1428, Argentina; bernardoelechner@gmail.com; 6 Instituto de Micología y Botánica (InMiBo), CONICET–Universidad de Buenos Aires, Buenos Aires 1428, Argentina; 7 Royal Botanic Gardens Victoria, Birdwood Avenue, Melbourne, VIC 3004, Australia; tom.may@rbg.vic.gov.au

**Keywords:** Basidiomycota, ectomycorrhizal fungi, hydnangiaceae, molecular phylogeny, taxonomic revision

## Abstract

The taxonomy of *Laccaria laccata*, the type species of the genus *Laccaria*, has long been ambiguous due to the absence of a reference sequence and the reliance on early, morphology-based descriptions. To resolve this issue, we selected a Code-compliant lectotype for *Agaricus laccatus*—the basionym of *L. laccata*—from Schaeffer’s 1762 illustration cited in Fries’ sanctioning work. Given the limitations of this historical material for modern species interpretation, we also designated an epitype based on Singer’s collection C4083 (BAFC) from Femsjö, Sweden, which was previously but not effectively designated as the “lectotype” by Singer. This epitype is supported by detailed morphological descriptions, iconography, and newly generated nrITS, nrLSU, and *RPB2* sequences, which have also been newly obtained from additional collections. Phylogenetic analyses consistently place the epitype of *L. laccaria* within a well-supported clade, herein designated as/*Laccaria laccata*, which includes sequences previously reported as falling within the “proxima 1 clade”. This integrative approach, combining historical typification with modern molecular and morphological data, stabilizes the nomenclature of *L. laccata* and provides a robust foundation for future studies of this ecologically and economically important genus of ectomycorrhizal fungi.

## 1. Introduction

*Laccaria laccata* serves as the type of the genus *Laccaria*, which belongs to the family Hydnangiaceae and includes species that are widely distributed across both hemispheres [1,2]. These fungi are known for their ability to form ectomycorrhizal symbioses with a remarkably diverse array of plants [3]. This diversity includes towering gymnosperms, such as Douglas fir (*Pseudotsuga menziesii*) along the Pacific coast of the US, to eucalyptus (*Eucalyptus*) species in southern Australia and the shortest known trees, such as dwarf willows in the genus *Salix*, which thrive in high-altitude alpine regions and Arctic zones [2]. Numerous studies have demonstrated the beneficial impact of species of *Laccaria* on plant growth [4], and their potential role in disease management [5], highlighting their importance in various natural ecosystems [6,7]. The ease of cultivating *Laccaria* mycelium in laboratory settings, along with its capacity to establish mycorrhizal associations under controlled conditions, has made this genus a prominent model for studying ectomycorrhizae [8]. Moreover, certain species of *Laccaria* are edible and frequently available in local markets such as in China [9,10]. According to He [11], approximately 85 species had been described by 2019, although Wilson [1] identified at least 116 recognized species (many formally unnamed) based on a phylogenetic species concept.

As discussed by Wilson [2], *Laccaria* exhibits a significant number of cryptic species that overlap in morphological traits, highlighting the need to apply the concept of phylogenetic species to achieve accurate identification. The current systematic framework for *Laccaria* is notably hindered by the absence of reference sequences for *L. laccata*, the type species of the genus.

This situation raises the possibility that many studies on ecological, biological, and technological aspects of *L. laccata* may have been based on misidentifications of other species. An analysis conducted in this study (data not published), utilizing nrITS sequences deposited in GenBank under the name *L. laccata*, revealed nearly 30 distinct potential terminal taxa through maximum likelihood (ML) analysis, highlighting the need to clarify the true boundaries of this species. *Laccaria laccata* was originally described by Scopoli in 1772 [12] under the name *Agaricus laccatus* Scop. The original description, based on collections from Idriam (Slovenia), is concise and relies solely on macromorphological characteristics, as detailed below: original text “Color pallide laccae, Lamellae remotae, continuae, apice quandoque ramosae. Habitat circa Idriam. Pileus convexus, non striatus. Lamellae crassisculae, in adulto fungo subdecurrentes. Stipes longus, calami crassitie, nudus, fistulosus, solitarius.” (text translated from Latin “Pale laccate colour. Lamellae distant, straight, irregularly eroded margin. Found near Idria. Pileus convex, not striate. Lamellae thick, somewhat decurrent. Stipe long, cylindrical, naked, hollow, solitary”). Subsequently, this binomen was sanctioned by Fries (1821: 106; Figure 1) [13] and was placed within “Trib. VIII. CLITOCYBE, Subtrib. 4. OESYPII”. Fries describes *A. laccatus* with a brief text: “gregarius, pileo subcarnoso tenaci squamuloso expallente, dein disco depresso, lamellis subdecurrentibus distinctis distantibus, stipite tenaci elongato” (text translated from Latin: “gregarious, with a subfleshy, tough, scaly-squamulose, fading pileus, then with a depressed disc, distinct and spaced lamellae subdecurrent on a tough, elongated stipe”).

**Figure 2:**
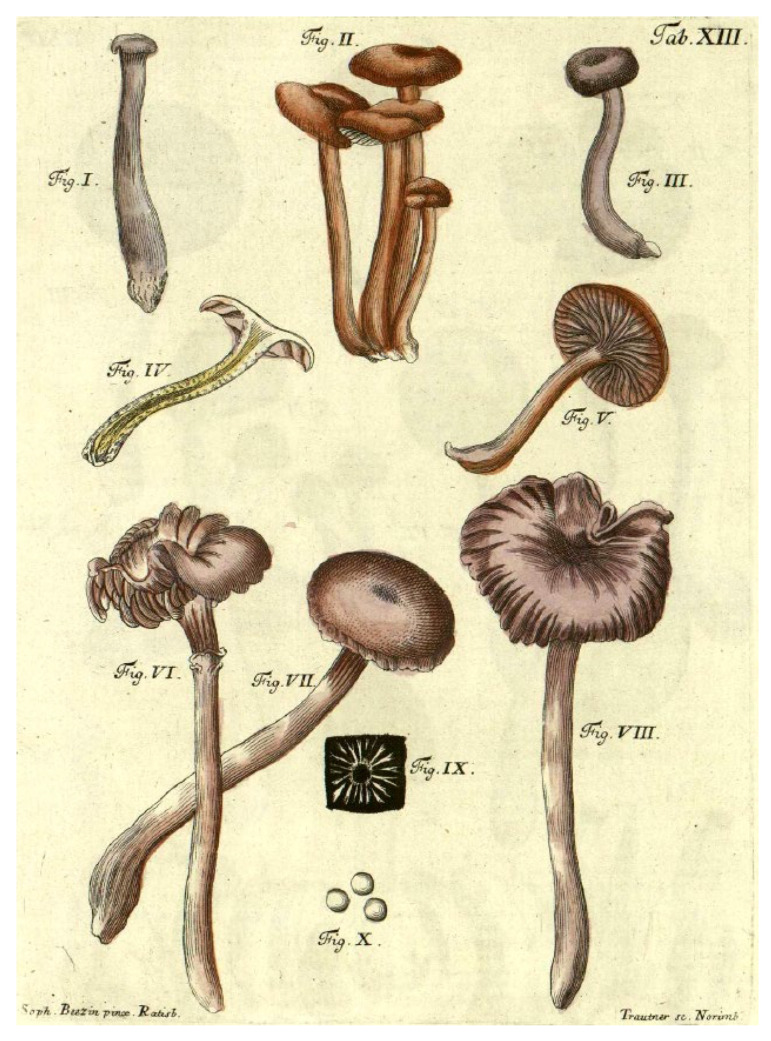
Table XIII of Schaeffer’s Fungorum qui in Bavaria et Palatinatu circa Ratisbonam nascuntur icones (https://bibdigital.rjb.csic.es/viewer/10986/#page=49&viewer=picture&o=bookmark&n=0&q= (accessed on 10 April 2023)).

Although Fries does not offer a microscopic description of *A. laccatus*, on page 107, he cites plate number 13 (Figure 2) [14], immediately after the reference to Scopoli’s name *A. laccatus*, before dealing with the varieties within *A. laccatus*. Plate 13 of Schaeffer [14] is not provided with a Latin binomial, but in the commentary on Schaeffer’s plates provided by Persoon in the revised posthumous edition [15] the plate was identified as *A. laccatus*.

Schaeffer’s plate includes ten figures: eight of these display basidiomata, while fig. ix depicts a white spore print, and fig. x illustrates spherical spores without evident spinules. It should be noted that in the plates by Schaeffer published in “Fungorum qui in Bavaria et Palatinatu…” spore ornamentation is not emphasized even in other taxa that are now known to have ornamented spores (e.g., *Cortinarius* and *Russula*).

**Figure 3:**
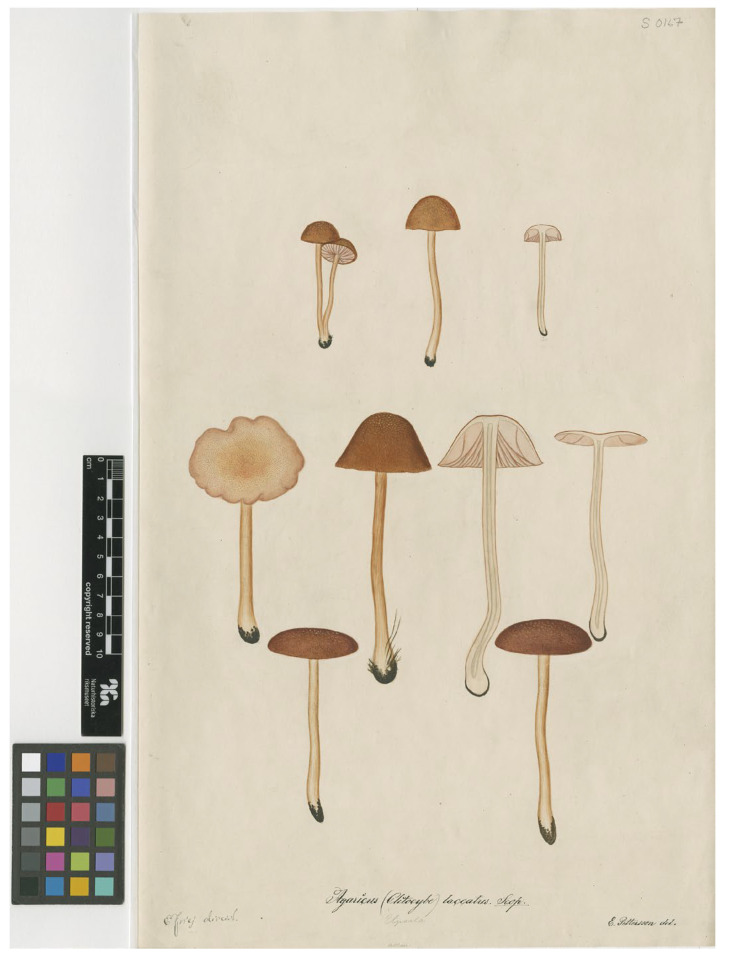
Plate S0167 of *Agaricus laccatus*, depicted by E. Pettersson in 1851 under the supervision of Fries (https://herbarium.nrm.se/specimens/S0167 (accessed on 10 April 2023)).

Fries considered two varieties under *Agaricus laccatus*, neither formally named: the first (a) with “rufo” or “carneo” pileus and “sicco subochraceo” and the second (b) with “pileo amethystino, sicco canescente”. The latter probably corresponds to the species that today we consider to be *Laccaria amethystina* (Huds.) Cooke. This demonstrates that Fries himself considered *Laccaria laccata* as an extremely variable species. In 1851, several years after the sanctioning publication *Systema mycologicum*, a plate of *A. laccatus* was prepared by E. Pettersson under the supervision of Fries (Figure 3).

**Figure 4:**
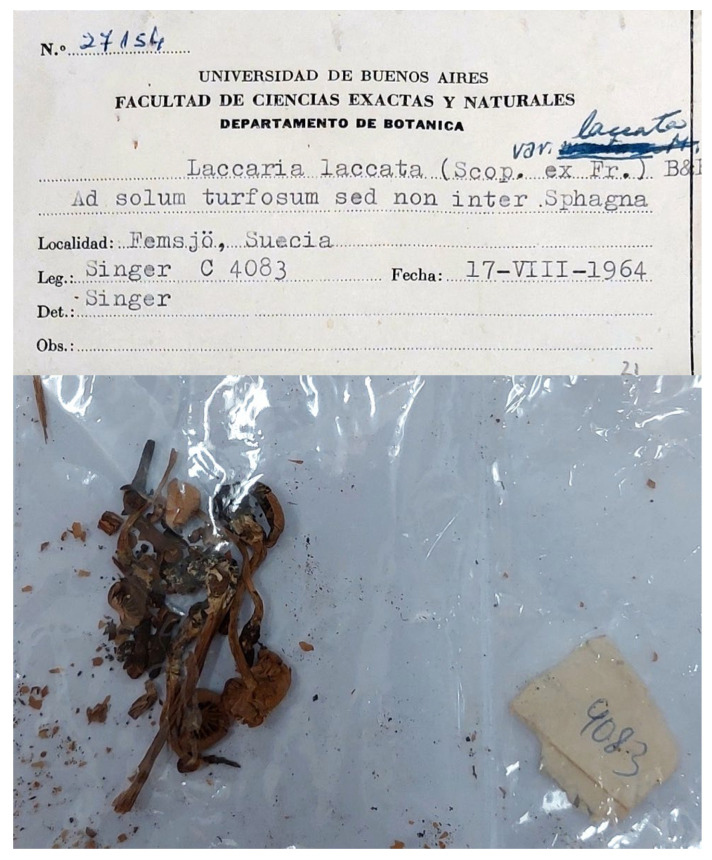
Specimen designated by Singer (C4083, BAFC) as the “lectotype” of *L. laccata* [16], herein designated as the epitype of *L. laccata*.

In the last century, as mycological standards evolved, it became necessary to designate a type for *Laccaria laccata* to redefine it in accordance with modern concepts. Regarding the typification of *Laccaria laccata*, Singer [16] proposed a “lectotype” based on a collection made in 1964 from the Femsjö area in Sweden, where Fries conducted his research for many years. At the time, Singer [16] recognized five different morphotypes among material he had collected at Femsjö, and he identified four of these as, respectively, *Laccaria amethystina*, *L. bicolor* (Maire) P.D. Orton, *L. tetraspora* Singer, and *L. ohiensis* (Mont.) Singer (considered to have two-spored basidia). Following the “residue rule”, Singer [16] selected the remaining morphological variant, which apparently had not been previously described by other authors, and associated with this variant the specimen Singer C4083 (BAFC) (Figure 4), designated as the “lectotype” of *L. laccata*. According to Singer’s macroscopic description, *L. laccata* was characterized by a reddish-brown fibrillose pileus that becomes pale ochre upon desiccation, featuring a flattened centre that often appears depressed, pale pink lamellae and a fibrillose stipe exhibiting white mycelium at the base. Microscopically, it was identified by mainly broadly ellipsoid spores measuring 8.5–9.5 × 6.7–8 µm (with dimensions of 7.5–8.8 × 5.7–6.8 µm excluding ornamentation), filamentous cheilocystidia measuring 12–24 × 2.5–3.8 µm, and a habitat preference for areas near peat bogs, typically in association with birch trees [16]. Under the Code in operation at the time, the Montreal Code [17], the specimen selected by Singer [16] could not be a lectotype, as it was collected after the publication of the name. In the absence of a holotype designation by the author (as is the case with *Agaricus laccatus*), a lectotype ought to have been selected from the material (specimens or illustrations) associated with the original description. Mueller and Vellinga [18] noted that Singer’s typification of *L. laccata* satisfied the rules for designating neotypes established by the ICBN [19], and they cite Singer C4083 (BAFC) as the “neotype”.

However, a neotype can only be designated when no original material exists, and this was not established by either Singer [16] or Mueller and Vellinga [18]. When Singer [16] considered the typification of *Agaricus laccatus*, the starting point date for basidiomycete Fungi was 1821, but in the Sydney Code [19] the starting point date was changed to 1753, but with the *Systema mycologicum* and some other works of Fries as “sanctioning works”. The role of the sanctioning works in typification was clarified by inclusion of Article 9.10 in the Melbourne Code [20] which indicated that, for a sanctioned name, the type may be selected from the elements indicated either directly in the original protologue or as part of the sanctioning work. Despite this addition, there continued to be debate about the relative role of the protologue and the sanctioning works in the typification of sanctioned names (e.g., see proposals F-001 and F-002 by Parra and Zamora in Hawksworth [21]), which was settled by the addition of examples to Art. F.3.9 in the San Juan Chapter F of the Code, concerning the typification of names accepted in the sanctioning works [22]. Example 11 specifically demonstrates that previously designated “neotypes” for names that are sanctioned may not be Code-compliant if illustrations are cited in the sanctioning work, and these illustrations must be used for lectotypification. In cases where an illustration or specimen must be chosen as a lectotype, but where the connection between that lectotype and a particular species as currently understood cannot be made with certainty, an epitype may be designated. Epitypes are not to be chosen automatically just to provide a sequenced collection, but only where the lectotype is “demonstrably ambiguous” (Art. 9.9). While the Code (Art 9.10) allows for correction of terms used in typification, it is not possible to automatically change Singer’s “lectotype” to a “neotype” because there is original material that otherwise must be used to select a type. Likewise, Singer’s “lectotype” cannot be changed to an epitype, because this latter definition must support another type, such as a lectotype, and at the moment there is no *Code*-compliant lectotype available. Mueller and Vellinga [18] argue that Singer’s “lectotype” (which they cited as a “neotype”) does not accurately represent the species *L. laccata* due to its micromorphological characteristics, which are at the extreme limits for this taxon as they understood it. This discrepancy, coupled with varying interpretations of *L. laccata* and the existence of segregate taxa in the literature, has contributed to further confusion in the identification of this species. The objectives of this manuscript are to (1) select an appropriate *Code*-compliant lectotype for *A. laccatus* based on the material associated with the protologue and the sanctioning work, (2) establish if this lectotype is demonstrably ambiguous in relation to identifiability to currently known species, and if so, provide an epitype collected in Femsjö that is consistent with Fries’ interpretation in order to establishes the concept of the name, and (3) offer modern iconography, macro- and microscopic descriptions, and genetic characterization in order to provide a future tool for phylogenetic analyses and common barcoding techniques.

## 2. Materials and Methods

### 2.1. Morphology

The macroscopic descriptions presented in this study are based on observations of fresh material collected in Italy, Sweden, and the UK. The specimens were dried using an electric dryer set to 30 °C. Voucher specimens were deposited in the herbarium Museo Civico di Storia Naturale Giacomo Doria (GDOR) of Genova (Italy). Descriptions of the micro-morphological characteristics were derived from the examination of both fresh and dried materials. For the dried samples, sections of the specimens were rehydrated in 5% aqueous potassium hydroxide (KOH) and stained with 1% Congo Red reagent. For ultramicroscopic studies, a lamella was placed on a 2 × 2 cm square of Whatman filter paper, folded, and stapled to retain spores. The specimen was rehydrated in concentrated ammonium hydroxide, dehydrated in 70% ethanol, fixed in ethylene glycol dimethyl ether, immersed in pure acetone, and then critically point dried and sputtered with gold–palladium. The details regarding the methodology used for optical and scanning electron microscopy (SEM) analyses are provided in Dovana and by Moreno [23,24].

A minimum of 30 basidiospores were measured for each collection. Basidiospore dimensions (measured without ornamentation) are expressed as (a) b–c–d (e), where (a) = minimum value, b = average − standard deviation, c = average, d = average + standard deviation, and (e) = maximum value. [x/y/z] indicates that altogether x spores, from y samples, from z collections were measured. Q indicates the quotient of length and width of the basidiospores in side view. Basidia were measured without sterigmata.

### 2.2. Molecular Analysis

Genomic DNA was extracted from dried fragments of eleven specimens using at least one of three methods: the sodium hydroxide-based method described by [25], Qiagen Mini Kit following the manufacturer’s instructions, or a CTAB method developed for RNA extraction with minor modifications [26]. The latest is the procedure that gave us the highest quality DNA and is outlined below.

Ten mg of dried mycelium was ground in LN2 with sterile micro pestle and microcentrifuge tubes. A total of 700 μL of pre-heated 65 °C extraction buffer, 1 μL of proteinase K, and 1 μL of RNAse were added to each microcentrifuge tube, mixed by inversion, and incubated for 15 min at 65 °C. After this incubation time, 700 μL of Chloroform:Isoamyl alcohol 24:1 (CHISAM) was added to each tube, mixing the solution by inverting the tubes. Each microtube was centrifuged at 4500 rpm for 20 min at room temperature. The supernatant was transferred into 2 mL tubes. This CHISAM wash step was then repeated again. After the second CHISAM wash, the supernatant was transferred into a clean 2 mL tube and ¼ volume of LiCl 10 M was added to each one. The microtubes were then placed into an ice filled cold box, and DNA was precipitated overnight at 4 °C. The following morning, the microcentrifuge tubes were centrifuged for 30 min at 4 °C at 10,000 rpm. The supernatant was completely removed, and the pellet was resuspended with 700 μL of pre-heated SSTE buffer at 60 °C. The microtubes were incubated for 5 min at 60 °C, until the pellet was completely resuspended. Two CHISAM washes were then performed. After the second supernatant collection, 2 volumes of pure ice-cold ethanol were added. The samples were then let rest in a −80 °C fridge for 1 h. After this precipitation, microtubes were centrifuged at 4 °C for 30 min at 10,000 rpm. Finally, the pellets were washed with 700 μL of 70% ice-cold ethanol, centrifuging the samples at 4 °C for 2 min at 10,000 rpm for each wash step. The pellet was then air-dried, helping the process by pipetting the liquid away. The pellet was then resuspended in 50 μL of DEPC H_2_O. For C 4083 samples, our first attempt to obtain a barcode for the ITS1 sequence consisted of a NGS-based approach. We used the template DNA extracted with the sodium hydroxide and Qiagen mini kit methods. The ITS1F/ITS4 primers were used with a combination of 4-degenerated bases at the beginning of the sequence, including Illumina stubs. A nested second round of PCR was applied to the amplicons in order to attach Illumina adapters to the amplicons. Sequencing was performed with an Illumina Miseq platform, manufactured by Illumina Inc. based in San Diego (CA, USA). After removing adapters from the demultiplexed raw-reads, we followed two approaches to obtain DNA barcodes. The first approach consisted in OTU clustering based on a 98% similarity threshold. The OTU representative sequences were then classified with BLAST (https://blast.ncbi.nlm.nih.gov/Blast.cgi, accessed on 1 January 2025), retaining those that matched with *Laccaria* sp. The second approach consisted in mapping the paired-end row reads on the genome of *Laccaria laccata* (Gen bank accession number). The mapping was performed with Hisat2 v2.2.1 [27]. The plasmid genomic sequence was included in order to capture the ITS DNA barcode sequence. Besides the NGS-based sequencing attempt described above, the internal transcribed spacer (nrITS) was initially amplified with primers ITS1F/ITS4 [28,29], a portion of the large subunit ribosomal ribonucleic acid (nrLSU) region was amplified with primers LR0R and LR5 [30], the RNA polymerase II subunit (RPB2) was amplified with the primers used in [31] and the translation elongation factor 1-alpha (TEF1-α) was amplified following the protocols reported in [32,33]. In cases where the above primer pairs did not yield a band or produced contaminated sequences, primers specifically designed for *Laccaria* were employed. These primers were designed in the present study using Primer3 v. 2.3.7 [34]. The selective primer sequences and amplification conditions are detailed in Table S1. PCR products were purified and sequenced by Sanger’s method. The DNA template was successfully amplified by using *Laccaria* specific primers and the following optimized PCR conditions. An initial denaturation temperature equal to 95 °C was applied for 1:00. This was followed by a denaturation step at 94 °C for 1:00, an annealing step of 55 °C for 0:30, and an elongation step of 60 °C for 1:00. This cycle was repeated 35 times. Lastly, a final extension step was then performed by applying 70 °C for 5:00. These PCR conditions were applied to all primer sets. The only difference is for RPB2 annealing temperature, which was set at 62.4° C.

### 2.3. Sequence Alignment, Dataset Assembly and Phylogenetic Analysis

New sequences generated in this study were checked and assembled using Geneious vs. R 11.1.5 [35] and compared to those available in the GenBank database (https://www.ncbi.nlm.nih.gov/genbank/) (accessed on 25 February 2025) by using the BLASTN algorithm.

The nrITS, nrLSU, *RPB2*, and *TEF1-α* datasets comprise sequences of *Laccaria* obtained from both the Northern Hemisphere and tropical regions, as documented by previous phylogenetic studies [1,13,36,37,38,39,40,41,42,43,44,45,46,47,48,49,50,51,52,53,54,55,56,57,58]. When available, holotype sequences were included (Table S2).

*Laccaria ambigua* K. Hosaka, A.W. Wilson and G.M. Mueller, which was reported as basal to the rest of the genus *Laccaria* in the phylogeny proposed by Wilson [1], was used as the outgroup. nrITS, nrLSU, *RPB2*, and *TEF1-α* sequences were independently aligned using MAFFT v 7.017 [59] with E-INS-i algorithm. To construct a phylogenetic tree, Maximum Likelihood (ML) was inferred with IQ-TREE 2 [60]. The best models were selected using ModelFinder [61] in IQ-TREE. The SH-like approximate likelihood ratio test (with 1000 replicates) and ultrafast bootstrap approximation (UFB) [62] (1000 replicates) were used to evaluate the reliability of clades.

Significant support was considered to be ≥80% for the SH-aLRT test and ≥95% for ultrafast bootstrap support in the ML analysis. The list of sequences used in our dataset is provided in Table S2.

### 2.4. Estimation of the Distribution of Laccaria laccata Using the nrITS Region

In order to provide an initial estimate of the distribution of *Laccaria laccata*, we conducted a BLAST analysis of an nrITS sequence of *Laccaria laccata* and selected sequences with an identity ranging from 100% to 99.5%. The list of sequences is presented in Table S3.

## 3. Results

### 3.1. Molecular and Phylogeny

**Figure 5:**
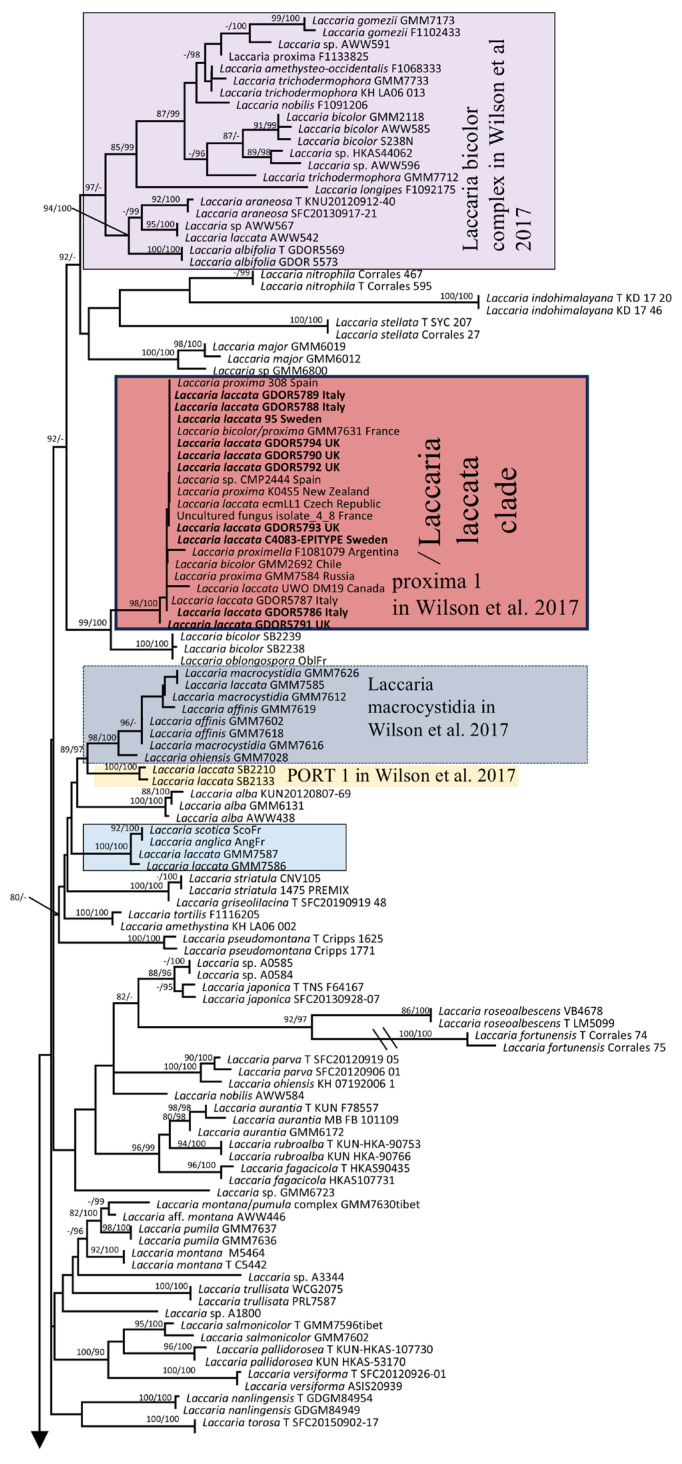

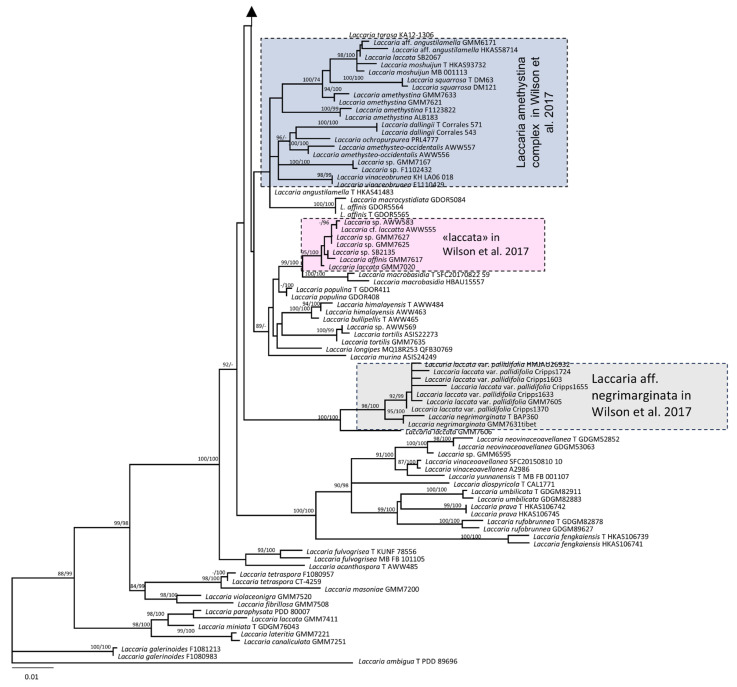
Maximum Likelihood phylogram obtained from the concatenated alignment of the nrITS, nrLSU, *TEF1-α* and *RPB2* loci of selected *Laccaria* species from the northern hemisphere. *Laccaria ambigua* (T PDD 89696) was used as an outgroup taxon. Values above or below branches indicate. bootstrap proportions SH-aLRT support ≥ 80% ultrafast bootstrap support ≥ 95%. The name of the clades refers to [1].

Ten nrITS, five nrLSU, and seven *RPB2* sequences of *Laccaria* were newly generated for this study; however, no *TEF1-*α sequence data were obtained. The nrITS, nrLSU, *RPB2*, and *TEF1-α* combined datasets comprised 3044 characters and 202 lines. A Maximum Likelihood tree with SH-aLRT and UFB values is shown in Figure 5. The present tree includes 19 sequences identified as *L. laccata*. Among these, ten sequences generated in this research, including the epitype of *L. laccata*, cluster with an additional nine sequences identified as *L. laccata*, *L. proxima*, and *L. bicolor* within a clade designated here as /*Laccaria laccata*. This clade corresponds to the clade labelled “proxima 1” in [1]. The remaining sequences of *L. laccata* are distributed across seven distinct positions or clades: *L. laccata* AWW542 groups with the “Laccaria bicolor complex”; *L. laccata* GMM7585 is positioned in the “Laccaria macrocystidia” clade; *L. laccata* SB2133 and SB2210 belong to the “PORT 1” clade; *L. laccata* GMM7586 and GMM7587 are situated in an unnamed clade; *L. laccata* SB2067 is affiliated with the *L. amethystina* complex; *L. laccata* GMM7606 is sister to the clade containing *L.* aff. *negrimarginata*; and finally, *L. laccata* GMM7020 is found within the “laccata” clade. The naming of the clades follows the classification delineated in [1]. Notably, the clade referred to as “laccata” in Wilson [1] is positioned as a sister group to *L. macrobasidia* and is independent from the /*Laccaria laccata* clade identified in the present study.

### 3.2. Morphology

**Figure 6:**
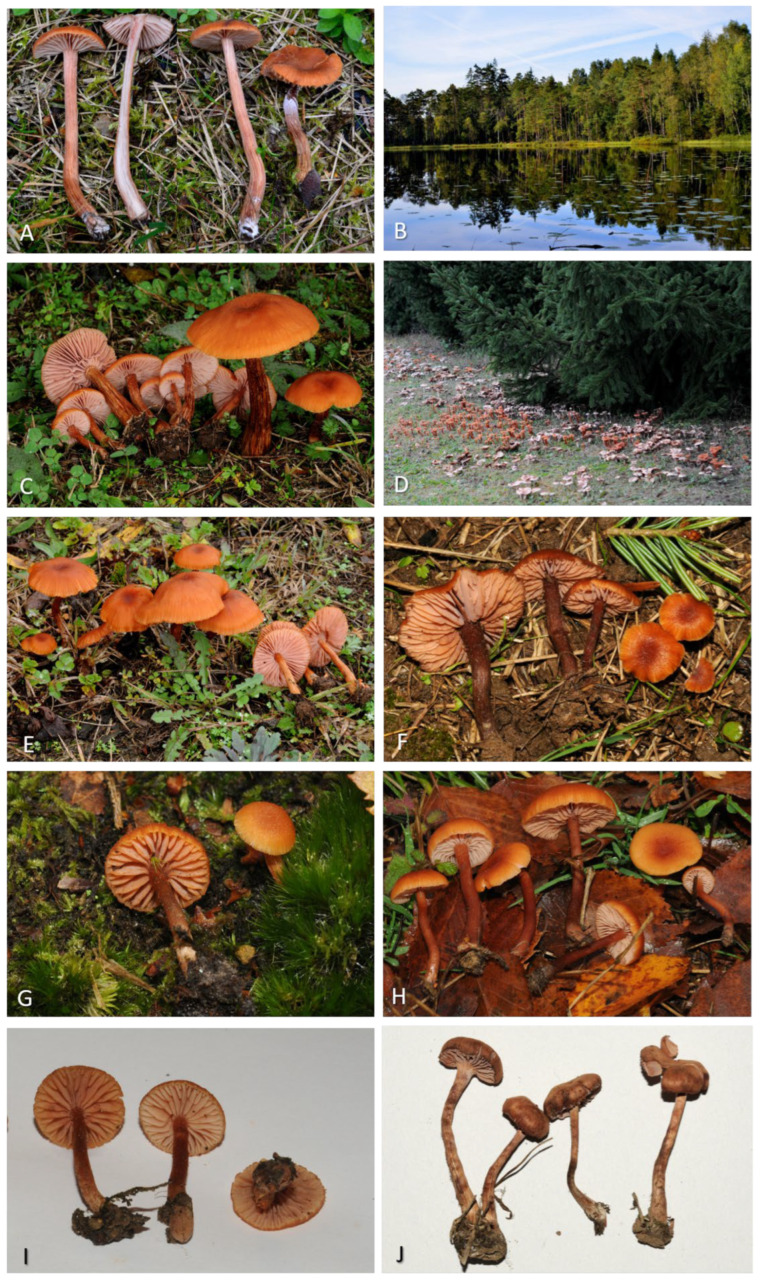
(**A**,**C**,**E**–**H**) Fresh basidiomata of *Laccaria laccata* (**A**) GDOR5795; (**C**) GDOR5788; (**E**) GDOR5787; (**F**) GDOR5792 (**G**) GDOR5794; (**H**) GDOR5791; (**I**) GDOR5790; (**J**) GDOR5786; (**B**) Sweden, Femsjö, Typical growth habitat of *Laccaria laccata*; (**D**) Italy, Borgo d’Ale, where MCVE and MCVE specimens were collected.

**Figure 7:**
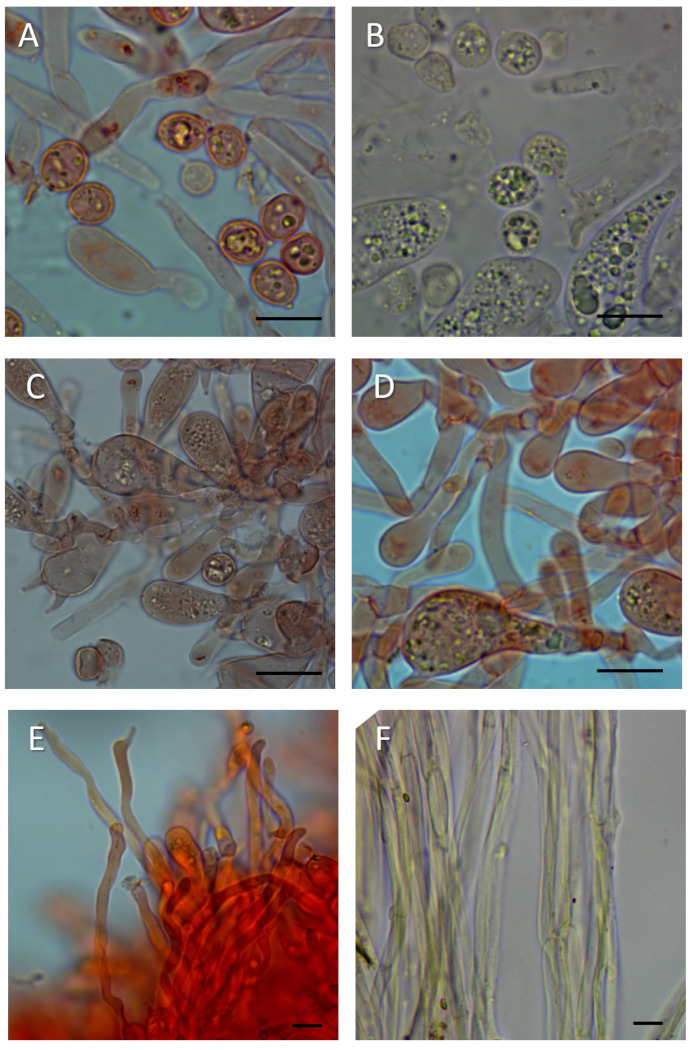
*Laccaria laccata* (epitype, C4083, BAFC): (**A**,**B**) basidiospores, (**C**,**D**) basidia and basidioles, (**E**) lamellar edge, (**F**) elements of stipitipellis. (**A**,**C**–**E**) in Congo red; (**B**,**F**) in water. Scale bars: 10 μm.

**Figure 8:**
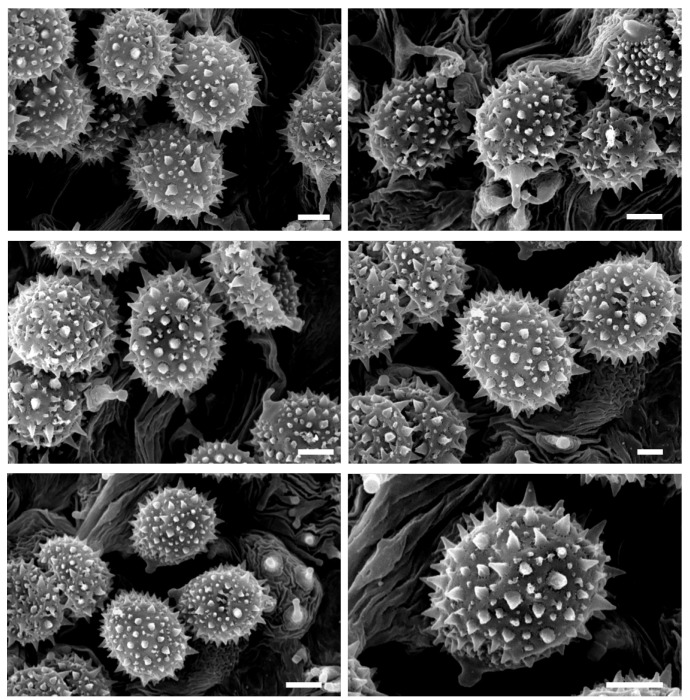
*Laccaria laccata* epitype. Spores (SEM photographs) (C4083, BAFC). Scale bars: 2 μm.

*Laccaria laccata* (Scop.) Cooke, Grevillea 12 (no. 63): 70 (1884) (Figure 6, Figure 7 and Figure 8).

Basionym: *Agaricus laccatus* Scop., Fl. carniol., Edn 2 (Wien) 2: 444 (1772), nom. sanct., Fr., Syst. mycol. 1: 106 (1821).

Lectotypus of *Agaricus laccatus* (designated here): illustration in Schaeffer (1762) [14] Fungorum qui in Bavaria et Palatinatu circa Ratisbonam nascuntur icones nativis coloribus expressae (Ratisbonae) Volume 1: tab. XIII, Fig. ii; MycoBank MBT 10026634.

Epitypus of *Agaricus laccatus* (designated here): Sweden, Femsjö, 17 August 1964, R. Singer, C 4083 (BAFC) [Previously designated as the “lectotype” in Singer (1967) [16]] MycoBank MBT 10026635; GenBank accession nrITS: PV700553, nrLSU: PV700591 and *RPB2*: PV835003.

Pileus 10–60 (–80) mm diam. is at first hemispheric then convex to plano-convex, generally with a central depression, with or without involute margin, very variable in different collections, rarely slightly striate, sometimes covered with fine squamules or tufts, rufus, flesh coloured vinaceous, orange to orange-brown, generally with darker centre; margin entire to undulate occasionally becomes eroded with age. Lamellae are close to distant, emarginate, sometimes with a decurrent tooth or decurrent, thin to thick, initially light pinkish flesh-coloured, then generally becoming dark pink-brown and white pruinose with age due to spore accumulation; the edge is entire and concolourous. Stipe 25–100 × 5–14 mm is cylindrical, to subclavate, sometimes with subbulbous base especially in larger basidiomata, solid, later fistulose, concolourous with pileus, fibrillose, from not striate to finely longitudinally striate, with white basal tomentum. Context hygrophanous is often lighter than basidioma surface. Smell resembles herbaceous-fungoid or subraphanoid. The taste is mild. The spore print is white.

Basidiospores (excluding ornamentation) (6.0–) 7.5–8.6–9.4 (–11.0) × (4.0–) 6.0–6.6–7.5 (–9.0) µm, Q = (1.00–) 1.19–1.30–1.41 (–1.65) [258/10/8] are generally from broadly ellipsoid to ellipsoid, echinulate; spines are 0.3–1.5 µm high, crowded. Basidia are 25–60 × 7.5–16 µm, clavate, hyaline, tetrasporic, very rarely bisporic. Pleurocystidia are not observed. Cheilocystidia are 15–70 × 3–8 µm, very variable in shape, cylindrical, flexuose to narrowly clavate, occasionally strangulate, rarely branched, absent or scattered to abundant, thin-walled, hyaline.

Pileipellis a cutis are made up of radially arranged cylindrical to subcylindrical hyphae, terminal elements from cylindrical, narrowly fusiform to clavate, contents hyaline to yellowish brown due to intracellular and sometimes with slightly encrusting pigment. Stipitipellis a cutis are of 5–10 µm wide hyphae with yellowish brown intracellular and slightly encrusting pigment.

Habitat and distribution: solitary to gregarious, terrestrial or on humus; present in both hemispheres, it is probably often identified as *L. proxima*, leading to an underestimation of its distribution. Based on our study of the nrITS region, *Laccaria* is distributed in Argentina, Canada, Chile, China, Czech Republic, Finland, France, Germany, India, Italy, Latvia, Lithuania, New Zealand, Poland, Russia, Slovakia, Spain, Sweden, United Kingdom, and the USA. *Laccaria laccata* can be associated with plants of *Abies* sp., *Betula pendula*, *Castanea dentata*, *Castanea sativa*, *Fagus* sp., *Nothofagus* sp., *Picea abies*, *Picea mariana*, *Pinus contorta* var. *latifolia*, *Pinus muricata*, *Pinus patula*, *Pinus radiata*, *Pinus sylvestris*, *Populus* sp., *Pseudotsuga menziesii*, *Quercus petraea*, *Quercus robur*, *Salix lapponum*, and *Tsuga canadensis*.

Specimens examined:

Italy: Piemonte, Fara Novarese, in broadleaf forest, 29 November 2014, leg. F. Dovana, GDOR5786; Piemonte, Vercelli, Borgo d’Ale, under *Picea abies*, GDOR5787, GDOR5788 and GDOR5789. Sweden: Småland, Femsjö, mixed forest, 08 September 2013, leg. F. Dovana, GDOR5795. United Kingdom: Kent, Bedgebury, mixed forest, 23 November 2019, GDOR5790, GDOR5791, GDOR5793, GDOR5793, F. Dovana; ibidem, under Abies alba, GDOR5792.

Note on *L. laccata* epitype:

For the optical and scanning electron microscopy (SEM) study of the epitype of *L. laccata* (C4083), small fragments of the basidioma were used to preserve its integrity. The analysis of sample C4083 has substantially confirmed what was previously reported by Singer [16]. In many spores, after the rehydration process, the spines were no longer visible under optical microscopy, but they could be easily observed only by SEM. The SEM analysis reveals spores that are subglobose to ellipsoidal in shape, with conical spines up to 1.5 µm in height. Some of the larger spines are surrounded by smaller spines, generally up to 0.3 µm in size (Figure 8). Below are reported the spore dimensions observed under optical microscopy after rehydration in potassium hydroxide: (7.0–) 7.5–8.2–9.0 (–9.5) × (5.5–) 6.0–6.7–7.5 (–8.0) µm, Q = (1.10–) 1.14–1.24–1.34 (–1.52). Numerous cheilocystidia were present on the lamellar edge.

## 4. Discussion

Fries’ interpretation in his sanctioning manuscript considered *A. laccatus* to be a taxon exhibiting extreme morphological variability, with colours ranging from pink to red to violet. In light of this variability, Fries’ concept of *A. laccatus* corresponds more closely to the modern definition of the genus *Laccaria* rather than to a single species. Plate XIII in Schaeffer’s work, as cited by Fries, displays eight images of basidiomata that could each pertain to a distinct species. The hypothesis that this plate includes multiple species was already indicated by Persoon in his commentary on Schaeffer’s plates [15].

Moreover, the same plate contains an image of spherical spores lacking spines; however, neither Fries, Schaeffer, nor, subsequently, Persoon refer to spore shape as a diagnostic character. Numerous monographs and dichotomous keys—developed even before the widespread adoption of molecular biology [3,16,18,63,64,65,66,67,68,69,70,71]—underscored the necessity of a detailed microscopic study for proper identification. Modern publications have further demonstrated that an integrated approach encompassing morphological, ecological, and genetic characters is essential for studying the genus *Laccaria*. For these reasons, the description provided in the sanctioning work and Schaeffer’s plate cannot offer an unequivocal modern interpretation of *A. laccatus*. Consequently, even the designation of a specific figure from the aforementioned plate as the lectotype must be backed by an epitype that integrates micro-morphological and molecular data. Although the typification process proposed by Singer in 1967 [16] does not conform to the current rules of the nomenclatural *Code*, his interpretation of *L. laccata* as a species with ellipsoid (rather than globose) spores has been established in the literature for over fifty years [63,64,65,66,67,68,69,70]. In some of these treatments, it is acknowledged that this ellipsoid-spored species is rare. Indeed, Mueller [3] and Mueller and Vellinga [18] treat *Laccaria laccata* as including *L. laccata* var. *pallidifolia* (Peck) Peck, a common variety with globose to subglobose spores, while the type variety is reported from “only a few localities in Europe and North America” [3] and not at all from the Netherlands [68]. Vesterholt [71] in *Funga Nordica* notes that var. *pallidifolia* [with mean Q = 1–1.1 (–1.15)] is “far more common” than var. *laccata* [with mean sp > 1.2] and goes as far as suggesting that “Singer’s 1967 typification of *L. laccata* with the rare element should be reconsidered”. We note also that in some treatments of *Laccaria* from Europe, among the 4-spored species that do not have violet colours, and discounting two *Salix*-associated species [*Laccaria montana* (from arctic to subarctic heathland) and *L. maritima* (in sand dunes)], there are only two species accepted, *Laccaria laccata* and *L. proxima*, as in Vellinga [68] and Vesterholt [71]. In contrast, other treatments, such as Ballero and Contu [65] recognise in addition to *L. laccata* and *L. proxima*, non-violet species such as *L. tetraspora* and *L. affinis*. If the typification of Singer is not taken up, there is no clear option as to which morphological concept the name *L. laccata* should be associated with.

Therefore, we consider it preferable to propose an epitype based on the specimen (C4083), which Singer [16] had previously designated as the “lectotype”. This approach avoids overturning a long-established interpretation of what is the type of the species and, thus, prevents further confusion. The decision to select this specimen as epitype was influenced by the successful acquisition of sequences from the ITS, LSU, and *RPB2* regions. These molecular data characterize the taxon phylogenetically and help resolve ambiguities that had previously arisen from the lack of reference DNA sequences.

In accordance with the decision to confirm Singer’s specimen as the epitype, Fig. ii from Schaeffer’s Plate XIII is here selected as the lectotype, as it is consistent with further modern collections that fall within the same phylogenetic species. It is necessary to select only one of the sporophores from the plate due to the high likelihood that the plate depicts multiple species. Another factor that needs to be taken into account is that there are significant differences in the colouring of the hand-coloured plates in the different editions of Schaeffer’s work. There were three editions subsequent to the original edition, which commenced publishing in 1762 [14]: the editio secunda of 1772; the editio tertia of 1780; and the editio nova of 1800 [72]. We have consulted on-line copies of the original edition and of the editio secunda and the editio nova. As well as slight differences between different editions and different copies of the same edition that may be due to the methods of preparing the image scans, the most striking difference is that while the original edition depicts on Tab. XIII, the figure chosen as the lectotype (fig. ii) with a darkish orange brown pileus, in contrast fig. i and iii have a distinct bluish or purple tint and other figures are redder or more violet in tone than fig. ii. In contrast, the *edition nova* depicts fig. ii as orange brown and the same for fig. vi, and then all other sporophores are depicted with a pinkish or red tone and no hint of purple or blue. Thus, across all editions, fig. ii is consistently depicted as orange brown, and this supports the choice to designate this particular figure as the lectotype. Schaeffer [14,72] does not mention colour in his description or in the caption to the plate, apart from indicating ‘*unicolor*’ [in contrast to other species that were bicoloured]. In volume four of Schaeffer’s work, where he applies the name *Agaricus laccatus* to Tab. XIII, he mentions the colour of the pileus as pallid purple with the stipe and lamellae as concolourous, while stating immediately after the colour that is was very changeable. However, among the synonyms cited, some are polynomials with the colour term rufous as part of the name. Persoon [15] specifically notes in his commentary that there is a high variation among the depicted figures, including in colour. He connects fig. i and iii with *Agaricus amethysteus,* noting the violet lamellae and stipe; he connects figs. ii, iv and v with *A. rosellus,* noting the subochraceous pileus, the rose-coloured lamellae tinged with flesh-colour and the concolourous stipe; and he connects fig. vi, vii and viii with *A. farinaceus*, noting the subochraceous pileus. It is not known how the plates in the *editio nova* of 1800, that was issued in concert with the commentary by Persoon [15], came to be coloured quite differently to the original edition, and in contrast to what is described by Persoon [15]. Whatever the reason, awareness of the variation in the colour of the sporophores depicted in the original plate necessitates choice of one figure. A further consideration in choosing fig. ii is that it substantially corresponds to Plate S0167 of *Agaricus laccatus* (https://herbarium.nrm.se/specimens/S0167; (accessed on 10 April 2023)), executed by E. Pettersson in 1851 under the supervision of Fries.

In this latter plate (Figure 3), ten basidiomata are depicted, each exhibiting a slender stipe and a pileus with an intense rufous colouration (with the exception of the leftmost specimen, which displays a faded cap) that shows fine squamulose features. All these characteristics are compatible with the concept of *L. laccata* as proposed by Singer and are observable in several basidiomata examined in the present study, which belong to the /*Laccaria laccata* clade as referred to in this study. *Laccaria laccata* as defined by the epitype and these recent collections is a small- to medium-sized species characterized by a pileus that displays colours ranging from various shades of bright red to orange or rufous (but never purple), smooth or ornamented with scales, and a stipe that is concolourous or sub concolourous, with smooth or markedly coarsely fibrillose surface sometimes with ± contrasting fibres. Microscopically, it exhibits broadly ellipsoid to ellipsoid basidiospores with densely conical spines with bases that do not exceed 1 µm in width, and filamentous cheilocystidia that are numerous or may be absent. Phylogenetic analyses place the epitype of *L. laccata* within a clade that includes sequences identified as *L. bicolor*, *L. proxima* and *L. proximella*. *Laccaria bicolor*, in its typical forms, is distinguished from *L. laccata* primarily by the presence of lamellae with lilac hues and violet mycelium at the stipe base [3,70]. According to several authors [3,67,70], *L. proxima* is differentiated from *L. laccata* mainly by its larger basidiomata and spores, although Mueller [3] noted that it is “sometimes difficult to differentiate *L. laccata* var. *laccata* from *L. proxima* without information on macromorphology”. Vesterholt in Funga Nordica [71] distinguished *L. proxima* by the presence of a scaly pileus and a stem coarsely fibrillose with ± contrasting fibres. In our study, we found basidiomes of *L. laccata* exhibiting characteristics previously attributed only to *L. proxima* (larger scaly pileus, stem coarsely fibrillose and larger basidiospores), raising the possibility that these two species, which were classified as separate by previous authors based on morphological features, may actually represent the same species from a phylogenetic perspective. Further studies that include sequenced type or references material of relevant species will be necessary to clarify the true taxonomic independence of *L. proxima* and *L. proximella* from *L. laccata* and from other related varieties and species. If indeed *L. laccata* and *L. proxima* are synonymous, the earlier name *L. laccata* would take precedence. Due to the widespread use of the name *L. laccata* over the more than 200 years since Scopoli [12], and in particular Fries [13], originally broadly characterised the species, any previous reports of *L. laccata* must be treated with caution unless sequenced voucher material is available.

## Figures and Tables

**Figure 1:**
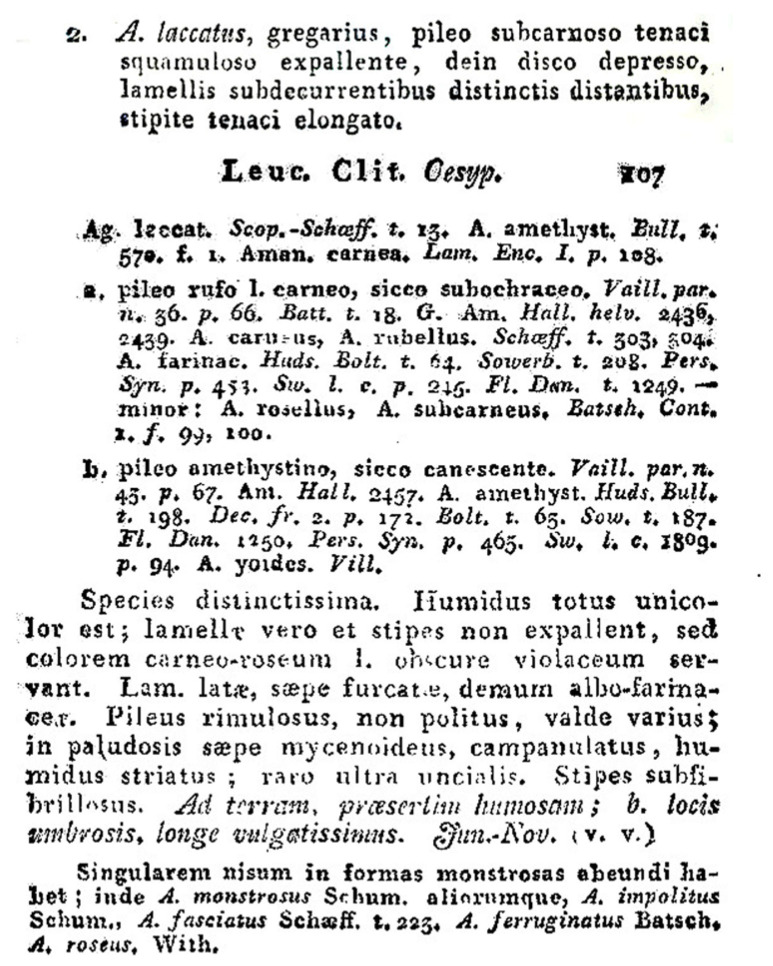
The treatment of the name *Agaricus laccatus* in the sanctioning work (Fries 1821: 106–107) (https://www.biodiversitylibrary.org/item/25498; accessed on 1 May 2025) [13].

## Data Availability

The original contributions presented in this study are included in the article/Supplementary Material. Further inquiries can be directed to the corresponding author.

## Supplementary Materials

The following supporting information can be downloaded at: https://www.mdpi.com/article/10.3390/jof11080575/s1, Table S1: Primers used and PCR conditions; Table S2: Sequences used in the phylogeny; Table S3: Selected sequences from the BLAST analysis of an nrITS sequence of *Laccaria laccata*. The sequences included in this table exhibit a sequence identity ranging from 100% to 99.5%.
