# Supplementary material for: Solving the Enigma of the Identity of Laccaria laccata
Source: J Fungi (Basel). 2025 Aug 1;11(8):575. doi: 10.3390/jof11080575 (PMC12387599; doi:10.3390/jof11080575)
Supplement: Supplementary file 1 [file jof-11-00575-s001.zip › 4. jof-3705502-Dichotomous Key to Red Species of Laccaria in Europe and North America.pdf]

Dichotomous Key to Red Species of *Laccaria* in Europe and North America

1a. Four-spored basidia

2a. Basal mycelium white

3a. Broadly ellipsoid to ellipsoid spores, red, red-orange, reddish brown to orange-brown pileus, pinkish flesh-colored lamellae, associated with both gymnosperms and broadleaf trees.

→ *Laccaria laccata* (including *L. proxima*)

3b. Globose basidiospores

4a. Reddish brown to orange-brown pileus, sporal spines exceeding 2 µm, in mixed forests in North America. → *Laccaria striatula*

4b. Brown–red to orange pileus often with wine red areas, sporal spines not exceeding 2 µm, associated with Salix spp. and Populus spp. (Salicaceae). → *Laccaria populina*

3c. Subglobose to broadly ellipsoid basidiospores

5a. Red brown or brick red to orange brown pileus, basal mycelium white, without wider cheilocystidia, associated with Salix spp. and *Dryas*, in alpine habitat. → *Laccaria montana*

5b. Reddish brown to vinaceous pileus, basal mycelium violet, presence of large filamentous to clavate cheilocystidia (31-92 x 5.5-11 µm), in sandy soil under Quercus virginiana. →*Laccaria vinaceobrunnea*.

3d. Oblong, subfusiform or fusiform-ellipsoid basidiospores with Q>1,4

6. Reddish orange-brown to dark reddish-brown pileus, oblong to subfusiform basidiospores, sporal spines not exceeding 0.5 µm, northern Europe, rare in eastern North America, found only in sand dunes or other very sandy areas, where they are putatively associated with species of *Pinus*. → *Laccaria maritima*

2b. Basal mycelium violet

7a. Reddish brown to brownish orange pileus, pinkish flesh-colored lamellae, subglobose to broadly ellipsoid basidiospores, spines 0,5-1,8 µm, associated with *Pinus* spp. in North America. → *Laccaria trichodermophora*

7b. Red-brown to greyish purple, brown or buff pileus, basal mycelium violaceous, subfusiform to fusiform-ellipsoid basidiospores, without sporal spines, present in sand dunes or very sandy soil; usually associated with species of Pinus, eastern and midwestern North America. → *Laccaria trullisata*

2c. Basal mycelium violet, soon becoming white

8. Reddish brown to brownish orange pileus, vinaceous to pinkish flesh lamellae, subglobose to broadly ellipsoid basidiospores with spines up to 1,5 µm, in North America under *Pinus* spp. And *Salix* spp.. → *Laccaria nobilis*

1b. Two-spored basidia, globose to subglobose basidiospores, basal mycelium white

9a. Rusty red-brown pileus, spore spines 1 - 1,8 µm, associated with plants belonging to the genus *Eucalyptus*. → *Laccaria fraterna*

9b. Red-brown or orange-brown pileus, spore spines 1,5-2 µm, in the alpine region associated with *Salix* spp.. → *Laccaria pumila*
