# Supplementary material for: Solving the Enigma of the Identity of Laccaria laccata
Source: J Fungi (Basel). 2025 Aug 1;11(8):575. doi: 10.3390/jof11080575 (PMC12387599; doi:10.3390/jof11080575)
Supplement: Supplementary file 1 [file jof-11-00575-s001.zip › Table S1.pdf]

Primer Sets				
	ITS	RPB2	LSU	TEF
	ITS_69_f & ITS_314_R	RPB2_298_F & RPB2_693R	LSU_L_6F & LSU LR	TEF_69_F & TEF_556_R
Forward sequence (5’->3’)	ATGTGGCTGTTAGCT GGCTT	GAGTATCTCAGGTCTTGA ACAGATACA	AGAGAAGTATTAT CCGCGCTGG	TCAACAAGATGGAC ACCACTAAGG
Reverse sequence (5’->3’)	AGAGCCAAGAGATCC GTTGC	GAGGTCAAATTGTCAAAA ATTGTCCAAATAAAT	TCTACCGCAGAAT CGTCACAAA	CTTGATGATACCAG TCTCAACACGAC
PCR Conditions				
Initial denaturation	95ºC, 1:00	95ºC, 1:00	95ºC, 1:00	95ºC, 1:00
Denaturation	94ºC, 1:00	94ºC, 1:00	94ºC, 1:00	94ºC, 1:00
Annealing	55ºC, 0:30	55ºC, 0:30	55ºC, 0:30	55ºC, 0:30
Extension	60ºC, 1:00	60ºC, 1:00	60ºC, 1:00	60ºC, 1:00
	Go to Step 2, 35x	Go to Step 2, 35x	Go to Step 2, 35x	Go to Step 2, 35x
Final extension	70ºC, 5:00	70ºC, 5:00	70ºC, 5:00	70ºC, 5:00
Hold	4ºC, infinite hold	4ºC, infinite hold	4ºC, infinite hold	4ºC, infinite hold
