# Supplementary material for: Solving the Enigma of the Identity of Laccaria laccata
Source: J Fungi (Basel). 2025 Aug 1;11(8):575. doi: 10.3390/jof11080575 (PMC12387599; doi:10.3390/jof11080575)
Supplement: Supplementary file 1 [file jof-11-00575-s001.zip › Table S2.pdf]

Taxa	Specimens	Locality	GenBank Accession number				References
			ITS	LSU	tef	rpb2	
Laccaria acanthospora	AWW485 (holotype)	China: Tibet	JX504102	JX504186	KU686073	KU685916	[1,39]
Laccaria aff. *angustilamella*	GMM6171	China	JX504132	_	_	_	[39]
Laccaria aff. *angustilamella*	HKAS58714	unknown	JX504168	JX504244	_	_	[39]
Laccaria affinis	GMM7618	France	KM067852	_	_	_	[42]
Laccaria affinis	GMM7619	France	KM067853	_	_	_	[42]
Laccaria affinis	GMM7617	France	KM067851	_	_	_	[42]
Laccaria affinis	GDOR5564	England: Kent	PQ642691	_	_	_	[23]
Laccaria affinis	GDOR5565 (epitype)	England: Kent	PQ642690	_	_	PQ653981	[23]
Laccaria affinis	GMM7602	France	KM067842	_	_	_	[42]
Laccaria alba	GMM6131	China	JX504131	JX504210	KU686079	KU685930	[1,39]
Laccaria alba	KUN 20120807-69	South Korea	MG519542	MG519583	MG551649	MG551616	[47]
Laccaria alba	AWW438	China	JX504094	JX504178	KU686072	KU685912	[1,39]
Laccaria albifolia	GDOR 5573	Italy	PQ642683	PQ642696	PQ653978	PQ653980	[23]
Laccaria albifolia	GDOR5569 (holotype)	Italy	PQ642680	PQ642694	PQ653979	_	[23]
Laccaria amethysteo-occidentalis	AWW557	USA: California	MT279220	MT279200	MT436061	MT431174	[51]
Laccaria amethysteo-occidentalis	AWW556	USA: California	JX504107	JX504191	_	KU685919	[1,39]
Laccaria amethysteo-occidentalis	F1068333	USA: Wisconsin	KU685629	KU685773	_	KU686061	[1]
Laccaria amethystina	GMM7633	France	JX504154	JX504228	_	_	[1]
Laccaria amethystina	GMM7621	France	JX504150	JX504224	KU686152	KU686046	[1,39]
Laccaria amethystina	KH LA06 002	USA: Louisiana	KU685759	KU685910	_	KU686059	[1]
Laccaria amethystina	F1123822	USA: Wisconsin	KU685760	KU685911	_	KU686071	[1]
Laccaria amethystina	ALB183	China: Tibet	JX504092	JX504176	KU686161	KU686058	[1]
Laccaria anglica	AngFr	France	GQ406459	_	_	_	[38]
Laccaria angustilamella	HKAS41483 (holotype)	China		JX504233	_	_	[39]
Laccaria araneosa	KNU20120912-40 (holotype)	Korea	MG519548	MG519588	MG551654	MG551621	[47]
Laccaria araneosa	SFC20130917-21	Korea	MG519549	MG519589	_	MG551622	[47]
Laccaria aurantia	KUN-F 78557 (holotype)	China	JQ670895	_	_	_	[40]
Laccaria aurantia	MB-FB-101109	China	JQ681209	_	_	_	[40]
Laccaria aurantia	GMM6172	China: Yunnan	KU685645	KU685789	_	_	[1]
Laccaria bicolor	AWW585	USA: Oregon	JX504111	JX504194	_	_	[39]

Laccaria bicolor	S238N	JGI/Genome	DQ179123	_	_	_	[1]
Laccaria bicolor	GMM2118	USA: California	KU685635	_	_	_	[1]
Laccaria bicolor	SB2238	Portugal	KM067892	KU685886	_	KU686030	[1]
Laccaria bicolor	SB2239	Portugal	KM067893	KU685887	_	KU686031	[1]
Laccaria bullipellis	AWW465 (holotype)	China: Tibet	JX504100	JX504184	_	KU685914	[1,39]
Laccaria canaliculata	GMM7251	Australia: Victoria	KU685669	KU685812	KU686090	KU685955	[1]
Laccaria cf *laccatta*	AWW555	USA: California,		KU685764	KU686074	KU685918	[1]
Laccaria dallingii	Corrales 543	Panama	MT279238	MT279213	MT436076	MT431187	[51]
Laccaria dallingii	Corrales 571 (holotype)	Panama	MT279240	MT279214	_	_	[51]
Laccaria diospyricola	CAL1771 (holotype)	India	MK776767	_	_	_	[53]
Laccaria fagacicola	HKAS90435 (holotype)	China	MW540806	_	_	_	[55]
Laccaria fagacicola	HKAS107731	China	MW540807	_	_	_	[55]
Laccaria fengkaiensis	HKAS106739 (holotype)	China	MN585657	MN621238	_	_	[52]
Laccaria fengkaiensis	HKAS106741	China	MN585658	_	_	_	[52]
Laccaria fibrillosa	GMM7508	New Zealand: Karamea	KU685706	KU685847	_	KU685989	[1]
Laccaria fortunensis	Corrales 74 (holotype)	Panama	MT279246	_	_	_	[51]
Laccaria fortunensis	Corrales 75	Panama	MT279247	_	_	_	[51]
Laccaria fulvogrisea	KUN-F 78556 (holotype)	China	JQ670896	_	_	_	[40]
Laccaria fulvogrisea	MB-FB-101105	China	JQ681210	_	_	_	[40]
Laccaria galerinoides	F1081213	Chile	KU685634	KU685778	KU686078	KU685929	[1]
Laccaria galerinoides	F1080983	Argentina	KU685632	KU685776	KU686077	KU685927	[1]
Laccaria gomezii	F1102433	CostaRica		MT279205	_	MT431180	[51]
Laccaria gomezii	GMM7173	CostaRica	MT279227	MT279207	MT436071	MT431182	[51]
Laccaria griseolilacina	SFC20190919-48 (holotype)	South Korea	MT322981	MT322983	MT333269	MT333266	[50]
Laccaria himalayensis	AWW484 (holotype)	China: Tibet	JX504101	JX504185	_	KU685915	[1,39]
Laccaria himalayensis	AWW463	China: Tibet	JX504098	JX504182	_	KU685913	[1,39]
Laccaria indohimalayana	KD 17-46	India	MK575505	_	_	_	[49]
Laccaria indohimalayana	KD 17-20 (holotype)	India	MK584157	_	_	_	[49]
Laccaria japonica	TNS-F64167 (holotype)	Japan	KU962988	_	_	_	[46]
Laccaria japonica	SFC20130928-07	Korea	MG519517	MG519565	MG551632	MG551594	[47]
Laccaria laccata	GMM7586	Russia	KM067835	KU685859	_	KU686000	[1,42]
Laccaria laccata	GMM7020	Russia	KU685652	KU685795	_	KU685938	[1]

Laccaria laccata	GMM7606	France	JX504147	JX504221	_	_	[39]
Laccaria laccata	GMM7585	Russia	KM067834	_	_	_	[42]
Laccaria laccata	GMM7587	Russia	KM067836	_	_	_	[42]
Laccaria laccata	SB2133	Portugal	KM067887	KU685884	KU686139	KU686027	[1]
Laccaria laccata	SB2210	Portugal	KM067890	KU685885	KU686141	KU686029	[1]
Laccaria laccata	SB2067	Portugal		JX504248	_	KU686026	[39]
Laccaria laccata	AWW542	USA: Illinois	KM067818				[42]
Laccaria laccata	GMM7411	Australia: Queensland	KU685690	KU685834	KU686108	KU685976	[1]
/Laccaria laccata clade (*)				_	_	_	
Laccaria proxima *	308	Spain	MN663149	_	_	_	Direct Submission
Laccaria proxima *	GMM7584	Russia	KU685717	KU685858	KU686120	KU685999	[1]
Laccaria proximella *	F1081079	Argentina	KU685633	KU685777	_	KU685928	[1]
Laccaria bicolor *	GMM2692 (F1077767)	Chile	KU685630	KU685774	_	_	[1]
Laccaria bicolor/proxima *	GMM7631	France	KM067858	KU685869	KU686129	KU686010	[1]
Laccaria laccata *	UWO:DM19 (MO 268112)	Canada	KY706162	_	_	_	[48]
Laccaria proxima *	K04S5	New Zealand	GQ267477	_	_	_	[37]
Laccaria sp. *	CMP2444	Spain	OR037584	_	_	_	Direct Submission
Uncultured fungus *	isolate_4_8	France	ON391386	_	_	_	Direct Submission
Laccaria laccata *	ecmLL1	Czech Republic	JX679364	_	_	_	Direct Submission
Laccaria laccata *	GDOR5786	Italy	PV700557	PV700594	_	_	this study
Laccaria laccata *	GDOR5787	Italy	PV700556	PV700593	_	_	this study
Laccaria laccata *	GDOR5788	Italy	PV700555	PV700592	_	PV835004	this study
Laccaria laccata *	GDOR5789	Italy	PV700549	_	_	_	this study
Laccaria laccata *	GDOR5790	England: Kent	_	_	_	PV835005	this study
Laccaria laccata *	GDOR5791	England: Kent	PV700550	_	_	PV835006	this study
Laccaria laccata *	GDOR5792	England: Kent	_	_	_	PV835007	this study
Laccaria laccata *	GDOR5793	England: Kent	PV700552	_	_	_	this study
Laccaria laccata *	GDOR5794	England: Kent	PV700554	_	_	PV835008	this study
Laccaria laccata *	GDOR5795 (Femsjo 95)	Sweden	PV700551	PV700590	_	PV835009	this study
Laccaria laccata *	(C4083, BAFC) (epitype)	Sweden	PV700553	PV700591	_	PV835003	this study
Laccaria laccata var. *pallidifolia*	GMM7605	France	JX504146	KU685901	KU686154	KU686048	[1,39]
Laccaria laccata var. *pallidifolia*	Cripps 1370	USA: Montana	DQ149849	_	_	_	[36]

Laccaria laccata var. *pallidifolia*	Cripps 1603	USA: Montana	DQ149851	_	_	_	[36]
Laccaria laccata var. *pallidifolia*	Cripps 1633	USA: Montana	DQ149853	_	_	_	[36]
Laccaria laccata var. *pallidifolia*	Cripps 1655	USA: Montana	DQ149847	_	_	_	[36]
Laccaria laccata var. *pallidifolia*	Cripps 1724	USA: Montana	DQ149857	_	_	_	[36]
Laccaria laccata var. *pallidifolia*	HMJAU26932	China	KM246792	_	_	_	Direct Submission
Laccaria lateritia	GMM7221	Australia: Victoria	KU685663	KU685806	_	KU685949	[1]
Laccaria longipes	F1092175	USA: Michigan	KU685637	KU685780	_	_	[1]
Laccaria longipes	MQ18R253-QFB30769	Canada	MN992191	_	_	_	Direct Submission
Laccaria macrobasidia	HBAU15557	Korea	MW871602	_	_	_	Direct Submission
Laccaria macrobasidia	SFC20170822-59	Korea	MT322982	MT322984	MT333268	MT333267	[50]
Laccaria macrocystidia	GMM7616	France	KM067850	KU685863	_	KU686004	[1,42]
Laccaria macrocystidia	GMM7612	France	KM067847	KU685861	KU686122	KU686002	[1,42]
Laccaria macrocystidia	GMM7626	France	KM067856	KU685865	KU686125	KU686006	[1,42]
Laccaria macrocystidiata	GDOR_5084	Italy	MW584896	_	_	_	[56]
Laccaria major	GMM6019	Costa Rica	KU685757	KU685908	KU686160	KU686056	[1]
Laccaria major	GMM6012	Costa Rica	KU685758	KU685909	_	KU686057	[1]
Laccaria masoniae	GMM7200	Australia: Victoria	KU685656	KU685799	KU686084	KU685941	[1]
Laccaria miniata	GDGM76043 (holotype)	China	NR_198640	OR785476	_	_	[57]
Laccaria montana	M5464 (Isotype)	Switzerland	OR419935	_	_	_	[58]
Laccaria montana	C5442 (holotype)	Switzerland	OR419936	_	_	_	[58]
Laccaria montana (aff.)	AWW446	France	JX504097	JX504181	KU686054	KU686157	[1,42]
laccaria montana/pumula complex	GMM7630tibet	China: Tibet	JX504151	JX504225	KU686009	KU686128	[1,39]
Laccaria moshuijun	HKAS93732	China	KU962989	_	_	_	[46]
Laccaria moshuijun	MB-001113	China	KU962985	_	_	_	[46]
Laccaria murina	ASIS24249	Korea	MG519552	MG519592	MG551658	MG551625	[47]
Laccaria nanlingensis	GDGM94954 (holotype)	China	OR689442	OR785478	OR826273	OR835198	[57]
Laccaria nanlingensis	GDGM84949	China	OR689441	OR785477	OR826274	OR835199	[57]
Laccaria negrimarginata	BAP360	China: Tibet	JX504120	_	_	_	[39]
Laccaria negrimarginata	GMM7631tibet	China: Tibet	JX504153	JX504227	KU686130	KU686011	[1,39]
Laccaria neovinaceoavellanea	GDGM52852 (holotype)	China	OR689447	OR785479	_	_	[57]
Laccaria neovinaceoavellanea	GDGM53063	China	OR689448	OR785480	_	_	[57]
Laccaria nitrophila	Corrales 467	Panama	MT279233	_	_	_	[51]

Laccaria nitrophila	Corrales 595 (holotype)	Panama	MT279236	MT279211	MT436074	MT431186	[51]
Laccaria nobilis	F1091206	USA: Michigan	KU685636	KU685779	_	_	[1]
Laccaria nobilis	AWW584	USA: Oregon	JX504110	JX504193	_	KU685922	[1,39]
Laccaria oblongospora	OblFr	France	GQ406466	_	_	_	[38]
Laccaria ochropurpurea	PRL4777	USA: Illinois	KU685733	KU685883	_	KU686025	[1]
Laccaria ohiensis	KH_07192006_1	USA: Indiana	KU685720	KU685871	_	KU686014	[1]
Laccaria ohiensis	GMM7028	Russia: Caucasus	KU685653	KU685796	_	KU685939	[1]
Laccaria pallidorosea	KUN-HKAS53170	China	MW540809	_	_	_	[55]
Laccaria pallidorosea	KUN-HKAS107730 (holotype)	China	MW540808	_	_	_	[55]
Laccaria paraphysata	PDD 80007	New Zealand	KM975424	_	_	_	Direct Submission
Laccaria parva	SFC20120919-05 (holotype)	Korea	MG519529	MG519573	MG551640	MG551604	[47]
Laccaria parva	SFC20120906-01	Korea	MG519527	MG519572	MG551639	MG551602	[47]
Laccaria populina	GDOR411 (holotype)	Italy	MN871894	MN873018	_	_	[54]
Laccaria populina	GDOR 408	Italy	MN871895	MN873017	_	_	[54]
Laccaria prava	HKAS106742 (holotpye)	China	MN585660	_	_	_	[52]
Laccaria prava	HKAS106745	China	MN585661	_	_	_	[52]
Laccaria proxima	F1133825	USA: Mississippi	KU685642	KU685786	_	KU686065	[1]
Laccaria pseudomontana	Cripps 1771	USA: Colorado	DQ149870	_	_	_	[36]
Laccaria pseudomontana	Cripps 1625	USA: Colorado	DQ149871	_	_	_	[36]
Laccaria pumila	GMM7637	France	JX504156	JX504229	KU686158	_	[1,39]
Laccaria pumila	GMM7636	France	KM067860	_	_	_	[42]
Laccaria roseoalbescens	LM5099 (holotype)	Mexico	KJ874328	KJ874331	_	_	[41]
Laccaria roseoalbescens	VB4678	Mexico	KJ590509	KJ590510	_		[41]
Laccaria rubroalba	KUN-HKA 90753 (holotype)	China	KX449358	_	_	_	[43]
Laccaria rubroalba	KUN-HKA 90766	China	KX449359	_	_	_	[43]
Laccaria rufobrunnea	GDGM82878 (holotype)	China	OR689443	OR785482	OR826272	OR835197	[57]
Laccaria rufobrunnea	GDGM89627	China	OR689444	OR785483	_	_	[57]
Laccaria salmonicolor	GMM7596tibet	China: Tibet	JX504143	JX504218	KU686151	KU686045	[1,39]
Laccaria salmonicolor	GMM7602	China: Tibet	JX504145	JX504220	_	_	[39]
Laccaria scotica	ScoFr	France	GQ406468	_	_	_	[38]
Laccaria sp.	A0584	Japan	KU685620	_	_	_	[1]
Laccaria sp.	A0585	Japan	KU685621	_	_	_	[1]

Laccaria sp.	GMM6723	China	KU685650	KU685793	_	KU685936	[1]
Laccaria sp.	AWW596	USA: Alaska	JX504116	JX504199	_	_	[39]
Laccaria sp.	A1800	Taiwan	KU685622	_	_	_	[1]
Laccaria sp.	F1102432	Costa Rica	KU685638	KU685781		KU686062	[1]
Laccaria sp.	GMM7167	Costa Rica	KU685655	KU685798	KU686083	_	[1]
Laccaria sp.	GMM7627	France		KU685866	KU686126	KU686007	[1]
Laccaria sp.	GMM7625	France		KU685864	KU686124	KU686005	[1]
Laccaria sp.	AWW583	USA: Oregon	KM067828	KU685768	_	_	[1]
Laccaria sp.	SB2135	Portugal	JX504172	JX504249	KU686140	KU686028	[1,39]
Laccaria sp.	GMM6595	China: Yunnan	KU685648	_	_	KU685934	[1]
Laccaria sp.	AWW591	USA: Oregon		KU685769	_	KU685924	[1]
Laccaria sp.	HKAS44062	China: Yunnan	JX504159	JX504235	_	KU686068	[1]
Laccaria sp.	AWW567	USA: Michigan	KM067824	KU685765	_	_	[1]
Laccaria sp.	GMM6800	Guatemala	KU685756	KU685907	KU686159	KU686055	[1]
Laccaria sp.	A3344	China	KU685627	_	_	_	[1]
Laccaria sp.	AWW569	USA: Michigan	JX504108	KU685766	KU685920	_	[1]
Laccaria squarrosa	DM63 (holotype)	Mexico	MF669958	MF669965	_	_	[45]
Laccaria squarrosa	DM121	Mexico	MF669960	MF669967	_	_	[45]
Laccaria stellata	SYC 207 (paratype)	Panama	KP877339	_	_	_	[44]
Laccaria stellata	Corrales 27	Panama	MT279231	MT279210	_	MT431185	[51]
Laccaria striatula	1475	USA: New York	OQ612526	_	_	_	Direct Submission
Laccaria striatula	CNV105	unknown	MT345281	_	_	_	Direct Submission
Laccaria tetraspora	F1080957	Argentina	KU685631	KU685775	_	KU685941	[1]
Laccaria tetraspora	CT-4259	Argentina	MH930294	_	_	_	Direct Submission
Laccaria torosa	SFC20150902-17 (holotype)	Korea	MG519561	MG519598	MG551664	MG551631	[47]
Laccaria torosa	KA12-1306	Korea	MG519562	_	_	_	[47]
Laccaria tortilis	ASIS22273	Korea	MG519533	_	_	_	[47]
Laccaria tortilis	F1116205	USA: Illinois	KU685641	KU685785	_	_	[1]
Laccaria tortilis	GMM7635	France	JX504155	KU685906	KU686156	KU686053	[1,39]
Laccaria trichodermophora	GMM7712	USA: Texas	KM067866	_	_	KU686012	[1,42]
Laccaria trichodermophora	GMM7733	USA: Texas	_	JX504230	_	KU686013	[1,39]
Laccaria trichodermophora	KH_LA06_013	USA: Louisiana	KM067881	KU685872	_	_	[1,42]

Laccaria trullisata	PRL7587	unknown	JX504170	JX504247	KU686153	KU686047	[1,39]
Laccaria trullisata	WCG2075	unknown	KM067894	_	_	_	[42]
Laccaria umbilicata	GDGM82883	China	OR689445	OR785485	OR826270	OR835194	[57]
Laccaria umbilicata	GDGM82911 (holotype)	China	OR689446	OR785486	OR826268	OR835192	[57]
Laccaria versiforma	SFC20120926-01 (holotype)	Korea	MG519556	MG519594	MG551660	MG551627	[47]
Laccaria versiforma	ASIS20939	Korea	MG519557	MG519595	MG551661	MG551628	[47]
Laccaria vinaceoavellanea	SFC20150810-10	Korea	MG519539	MG519580	MG551646	MG551614	[47]
Laccaria vinaceoavellanea	A2986	Korea	JN942810	JN939738	_	JN993520	Direct Submission
Laccaria vinaceobrunea	F1110429	USA: Texas	_	KU685783	_	_	[1]
Laccaria vinaceobrunea	KH_LA06_018	USA: Louisiana	_	KU685873	_	KU686015	[1]
Laccaria violaceonigra	GMM7520	New Zealand: Otago	KU685707	KU685848	_	KU685990	[1]
Laccaria yunnanensis	MB-FB-001107 (KUN-78558)	China	JQ670897	_	_	_	[40]
Outgroup							
Laccaria ambigua	PDD 89696	New Zealand	KU685725	KU685876	KU686132	KU686018	[1]
