# Supplementary material for: Solving the Enigma of the Identity of Laccaria laccata
Source: J Fungi (Basel). 2025 Aug 1;11(8):575. doi: 10.3390/jof11080575 (PMC12387599; doi:10.3390/jof11080575)
Supplement: Supplementary file 1 [file jof-11-00575-s001.zip › Table S3.pdf]

Taxa	GenBank ITS	Locality	Host
Laccaria bicolor	KU685630	Chile: Maullin	Pinus radiata
Laccaria bicolor	PP464389	Canada: Quebec	?
Laccaria bicolor	KM067858	France	Abies and Fagus
Laccaria bicolor	MN992606	Canada: Quebec	?
Laccaria cf. proxima	JN858078	USA	Pinus muricata
Laccaria laccata	JX679365	Czech Republic	Quercus sp.
Laccaria laccata	JX679364	Czech Republic	Quercus sp.
Laccaria laccata	GQ406464	France	?
Laccaria laccata	JX679363	Czech Republic	Quercus sp.
Laccaria laccata	EU819477	USA	Castanea dentata
Laccaria laccata	KY706162	Canada: Ontario	?
Laccaria proxima	AJ699073	India:Nilgiris	Pinus patula
Laccaria proxima	JX504142	France	?
Laccaria proxima	JX504152	France	?
Laccaria proxima	KX496976	China	?
Laccaria proxima	ON877167	China	?
Laccaria proxima	PP383813	Canada	?
Laccaria proxima	GQ406467	France	?
Laccaria proxima	JQ310816	USA	Pinus muricata
Laccaria proxima	DQ149852	USA	?
Laccaria proxima	MW376681	Spain: Galicia	Pseudotsuga menziesii
Laccaria proxima	OK346416	USA: Oregon	?
Laccaria proxima	ON129347	USA: New York	?
Laccaria proxima	PP464424	Canada: Quebec	?
Laccaria proxima	PP464540	Canada: Quebec	?
Laccaria proxima	DQ499639	Germany	?
Laccaria proxima	KU687391	Czech Republic	?
Laccaria proxima	OR765730	Germany: Welle	?
Laccaria proxima	DQ068958	Lithuania	Pinus sylvestris
Laccaria proxima	PQ644334	USA: Washington	?
Laccaria proxima	PV659608	USA: California	?
Laccaria proxima	PV644154	New Zealand	?
Laccaria proxima	PV708693	USA: Washington	?
Laccaria proxima	MH979277	USA: Wisconsin	?
Laccaria proxima	KM067833	Russia	Abies
Laccaria proxima	MT908294	Slovakia	?
Laccaria proxima	GQ267477	New Zealand	Pinus radiata
Laccaria proxima	MN663149	Spain	Castanea sativa
Laccaria proxima	JX907813	Latvia	Picea abies
Laccaria proxima	MF926547	Russia	Picea abies
Laccaria proxima	PV259572	Canada	Tsuga canadensis
Laccaria proxima	PQ652462	Sweden	?
Laccaria proxima	GU931707	USA: California	?
Laccaria proximella	KU685633	Argentina: Neuquen	Nothofagus sp.
Laccaria purpureobadia	PV051431	United Kingdom	?
Laccaria sp.	JX030273	USA: New York	?
Laccaria sp.	FJ168604	USA	?
Laccaria sp.	JX030274	USA: New York	Castanea dentata
Laccaria sp.	MH794937	Italy	Quercus robur

Laccaria sp.	KM576422	France	Q. petraea and Q. robur
Laccaria sp.	OQ555338	United Kingdom	Betula pendula
Laccaria sp.	OR037586	Spain	Quercus sp.
Laccaria sp.	OR037582	Spain	Castanea sp. and Betula sp.
Laccaria sp.	OR037583	Spain	Betula sp.
Laccaria sp.	OR037584	Spain	Pinus sylvestris
Laccaria sp.	OR037585	Spain: Los Arredores	Betula sp.
Uncultured ectomycorrhiza	EU427324	Finland	Picea abies
Uncultured ectomycorrhizal	AJ630020	Finland	Pinus sylvestris
Uncultured ectomycorrhizal	FN860027	Finland	Pinus sylvestris
Uncultured fungus	AB612229	Sweden	?
Uncultured fungus	MK490709	Poland	?
Uncultured fungus	KY088295	Lithuania	Pinus sylvestris
Uncultured fungus	MW214891	Lithuania	?
Uncultured fungus	KF156287	Sweden	Picea abies
Uncultured fungus	DQ414726	United Kingdom	Salix lapponum
Uncultured fungus	ON391386	?	Quercus petraea
Uncultured Laccaria	KJ591041	Germany	Populus sp.
Uncultured Laccaria	FJ197205	USA: California	Pine sp.
Uncultured Laccaria	KC702657	Canada	Picea mariana
Uncultured Laccaria	KC702663	Canada	Picea mariana
Uncultured Laccaria	KC702623	Canada	Picea mariana
Uncultured Laccaria	HM146831	?	Pinus sylvestris
Uncultured Laccaria	GU553370	USA	Castanea dentata
Uncultured Laccaria	KM008628	Canada	Pinus contorta var. latifolia
Uncultured Laccaria	KC702617	Canada	Picea mariana
Uncultured Laccaria	KC702655	Canada	Picea mariana
Uncultured Laccaria	KC702641	Canada	?
Uncultured mycorrhizal	EF195612	Germany	Pseudotsuga menziesii
